# Recruitment patterns of hothubs and dark functional networks correlating activity and connectivity with magnetoencephalography

**DOI:** 10.1038/s41598-025-34860-0

**Published:** 2026-01-16

**Authors:** Hao Xiong, Jin-Jin Chen, Chen-Guang Wang, Jing Gao, Qi-qi Chen, Feng Lin

**Affiliations:** 1 Department of Rehabilitation Medicine, Sir Run Run Shaw Hospital, Zhejiang University School of Medicine, 3 East Qingchun Road, Hangzhou, 310016 China; 2 Department of Cardiology, The Second Affiliated Hospital, School of Medicine, Zhejiang University, Hangzhou, 310000 China; 3 The First Affiliated Hospital of Nanjing Medical University, Nanjing, 210029 Jiangsu China; 4 Sir Run Run Hospital, Nanjing Medical University, Nanjing, 211100 Jiangsu China; 5 Department of Magnetoencephalography, Nanjing Brain Hospital Affiliated to Nanjing Medical University, Nanjing, 210029 Jiangsu China

**Keywords:** Functional brain networks, Hot hubs, Motor imagery, Dysphagia, MEG, Event-related (de)synchronization (ERD/ERS), Clinical trials, Neuroscience

## Abstract

In functional imaging studies, the activity and functional connectivity of regions of interest (ROIs) are two indexes to measure the importance of ROIs. This study aimed to investigate the activity-connectivity coupling relationships of ROIs during action observation-induced swallowing imagery and non-induced swallowing imagery tasks. Magnetoencephalography data were collected from fourteen healthy young participants. Time–frequency analysis of event-related spectral perturbations was conducted to assess the activity of ROIs during the tasks. Using corrected imaginary phase locking values to construct brain functional networks, network parameters are computed to assess connectivity. The correlation between activity and connectivity was evaluated using Pearson coefficients. Furthermore, the nodes were categorized into hot hubs (strong activity + strong connectivity), nonhub hotspots (strong activity + weak connectivity), cold hubs (weak activity + strong connectivity), and nonhub cold spots (weak activity + weak connectivity) for further analysis, and dark functional networks (subnetworks composed of cold hubs) were detected and analyzed. Both tasks exhibited positive activity-connectivity coupling relationships, however, the coupling strength remained below a moderate level (< 0.6). Notably, both tasks demonstrated limited activation of hot hubs. During the action observation-induced swallowing imagery task, there was a notable increase in the activation of non-hub hotspots within the visual system, extending bilaterally. Additionally, the dark functional network, composed of cold hubs, exhibited increased swallowing-related ROIs in action observation-induced swallowing imagery task. This study provides insights into the activity-connectivity coupling and dark functional network characteristics during swallowing imagery tasks. The findings contribute to our understanding of the neural mechanisms underlying swallowing imagery and may have implications for the development of therapeutic interventions for swallowing disorders.

**Supplementary Information:**

The online version contains supplementary material available at 10.1038/s41598-025-34860-0.

## Introduction

Action observation and motor imagery are established rehabilitation strategies for motor disorders, capable of enhancing motor function without physical execution by modulating activity in key brain regions^1–5^. In the context of swallowing, a complex sensorimotor behavior requiring precise bilateral cortical coordination^6–10^, these techniques can activate “hotspots”—highly active regions of interest—similar to those engaged during actual swallowing, thereby improving swallowing performance^11–13^. Concurrently, research on functional connectivity has highlighted the role of highly connected brain regions, or “hubs”, which are crucial for information processing. Swallowing tasks enhance connectivity within a network including the bilateral insula, sensorimotor, and premotor areas^14,15^, and optimizing this network’s efficiency can improve swallowing outcomes^16–18^.

However, the importance of brain regions can be assessed through two distinct dimensions: “activity” and “functional connectivity,” the relationship between which remains unclear. A pivotal question raised by Van den Heuvel and Sporns concerns the relationship between highly connected nodes and highly active nodes^19^. While it might be implicitly assumed that critically positioned hubs would necessarily coincide with highly active hotspots, emerging evidence reveals a more complex reality^20,21^. For instance, in a study on Broca’s aphasia, Lin et al. demonstrated that hubs are not invariably hotspots and emphasized the significance of a dark functional network—a subnetwork composed of cold hubs (nodes characterized by weak activity but strong connectivity). They proposed a taxonomy where nodes are classified into four types based on activity and connectivity: hot hubs (strong activity, strong connectivity), cold hubs (weak activity, strong connectivity), non-hub hotspots (strong activity, weak connectivity), and non-hub cold spots (weak activity, weak connectivity)^22^. Currently, there is a notable scarcity of evidence investigating the interplay between hotspots and hubs during swallowing tasks. As a midline motor behavior distinct from lateralized limb movements, swallowing necessitates the precisely orchestrated integration of numerous cortical and subcortical regions across both cerebral hemispheres. This characteristic offers a unique opportunity to examine whole-brain network coordination, thereby defining a critical knowledge gap that the present study is designed to address.

To address this gap, we employed both Non-Induced Swallowing Imagery (NISI) and Action Observation-Induced Swallowing Imagery (AISI) paradigms, with neural activity recorded using Magnetoencephalography (MEG). Regarding the paradigm, previous research and our pilot study^23^ suggest that combining action observation with motor imagery produces a synergistic effect, leading to more robust cortical activation than either approach alone^4,5^. Our primary objective was to systematically investigate the relationships between highly activated regions (hotspots) and highly connected regions (hubs) during NISI and AISI tasks. We hypothesized that highly active and highly connected brain regions would be distributed in distinct areas to subserve their respective functions in both tasks. Furthermore, we hypothesized that AISI, compared to NISI, would augment the recruitment patterns of both hotspots and hubs within the brain network, manifesting as a greater number of highly active brain areas and highly connected brain regions. This study aims to provide novel perspectives and analytical approaches for exploring brain functional network activation patterns during dynamic tasks.

## Materials & methods

### Participants

Fourteen healthy young participants (seven males and seven females) between the ages of 20 and 30 years were recruited for this study. The data of 12 participants were previously analyzed and published in the Brain Sciences journal^23^. For this study, we added two more participants and conducted further analysis based on the previously reported inclusion and exclusion criteria (Table 1)^24,25^.

**Table 1:** Inclusion and exclusion criteria.

Inclusion criteria	Exclusion criteria
Right handedness	Visual and visuospatial disorders
Age 20–30	Hearing impairment
MMSE > 24	Cognitive dysfunction or mental impairment
Average score of modified KVIQ-10 ≥ 2.5, and the swallowing imagery score ≥ 3	Contraindications of MRI, intolerance of 30 min MEG and 10 min MRI
	Respiratory or swallowing dysfunction

Visual and kinesthetic imagery is commonly used strategies for performing motor imagery tasks^26^. Their visual and kinesthetic imagery abilities were assessed using the kinesthetic and visual imagery questionnaire-10 (KVIQ-10)^27^. Since the existing imagery scales did not include an assessment of swallowing imagery, we added an item related to swallowing imagery to the KVIQ-10. Prior research has reported that kinesthetic imagery activates neural pathways more similar to actual motor execution^28^, therefore, we instructed the participants to perform the tasks using kinesthetic imagery as much as possible. The demographic characteristics of the participants are presented in Table 2 which includes the mean score of the modified KVIQ-10 and the visual and kinesthetic imagination scores related to swallowing. The results indicate that participants’ self-rated visual imagery scores were significantly higher than their kinesthetic imagery scores (*P* < 0.01).

**Table 2:** Subjects details.

ID	Age	Years of education	MMSE	Modified KVIQ-10	Visual imagination score of swallowing	Kinesthetic imagination score of swallowing
1	23	15	29	4.58	5	4
2	21	15	30	5	5	5
3	25	19	30	3.58	4	3
4	24	18	29	4.25	4	3
5	26	19	30	4.33	5	3
6	23	16	29	4.67	5	4
7	23	16	30	4.83	5	4
8	21	15	29	4.92	5	5
9	22	15	30	4.5	5	4
10	22	15	30	4.83	5	4
11	26	18	30	3.5	3	3
12	22	15	30	4.33	5	4
13	26	19	30	4.15	4	3
14	24	18	30	4.32	5	4

### Data acquisition

**Fig. 1:**
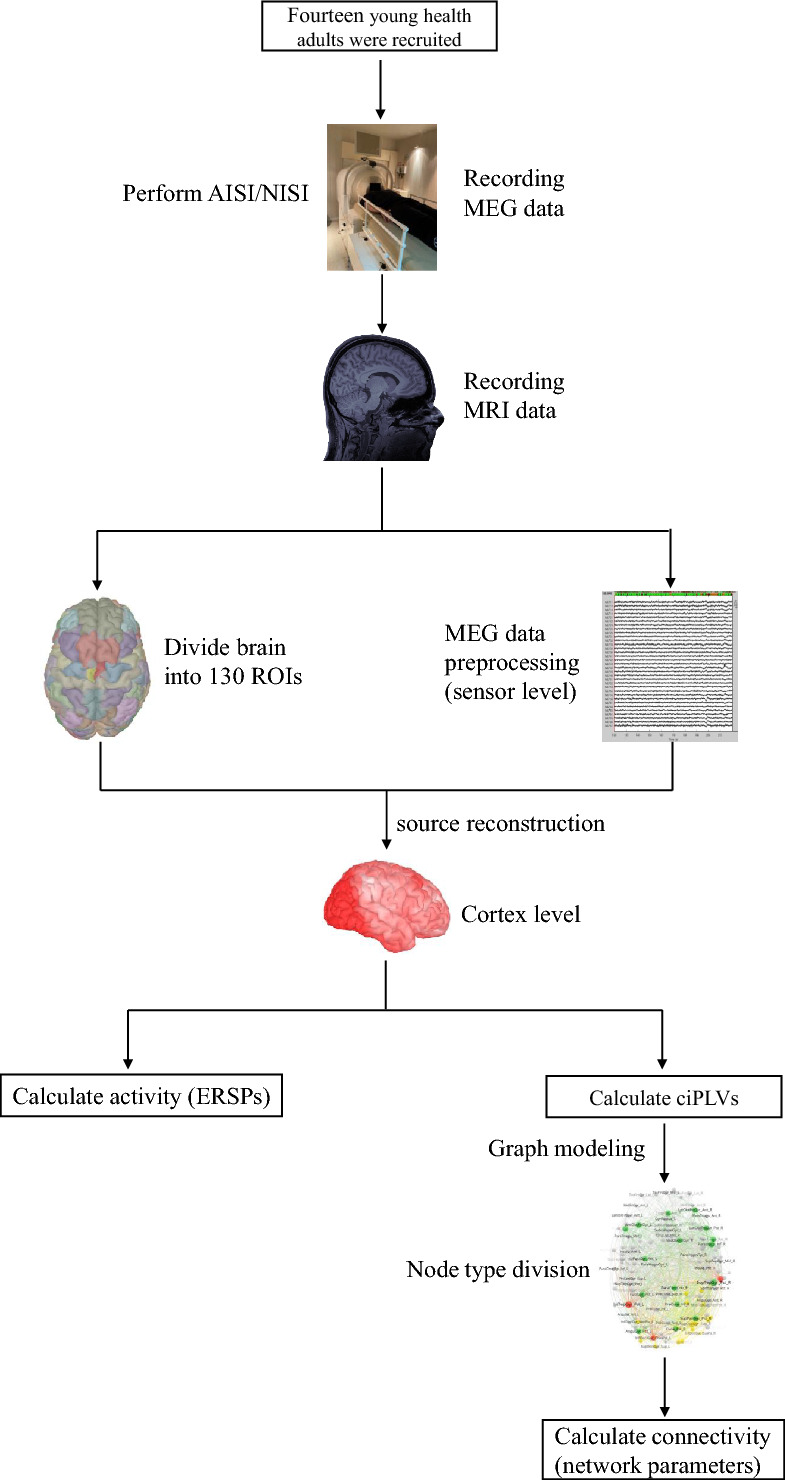
Experimental flow chart.

The data acquisition in this study have been described in a previous paper. For detailed information, please refer to Appendix 1 or the previously published paper^23^. Figure 1 is the flowchart of the experiment.

### Experimental paradigm

**Fig. 2:**
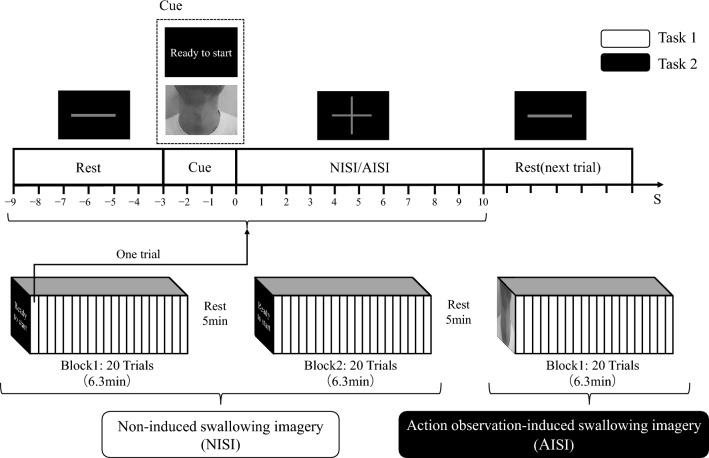
Experimental paradigm.

The experimental procedure consisted of two tasks: non-induced swallowing imagery (NISI) and action observation-induced swallowing imagery (AISI). For NISI (Task 1), participants completed two blocks, each consisting of 20 trials. Between the blocks, there was a 5-min resting period. The experimental process is illustrated in Fig. 2. Each trial began with a gray “-” picture displayed at the center of the screen for 6 s (s), during which participants were instructed to rest and observe the picture. Next, a picture of “ready to start” in Chinese was shown for 3 s as cue materials, indicating the start of the imagination task. Following that, a gray “ + ” picture was displayed for 10 s, during which participants were instructed to imagine swallowing while observing the picture. The trial ended with the display of the next gray “-“ picture, signaling the end of the trial and the beginning of the next one. Each trial lasted for a total of 19 s (6 s + 3 s + 10 s).

After a 5-min resting period following the completion of Task 1, participants proceeded to perform AISI (Task 2). Task 2 consisted of a single block with a total of 20 trials, as shown in Fig. 2. Instead of the picture of “ready to start”, swallowing videos were used in Task 2 as cue materials. The swallowing videos were close-up shots of swallowing actions of 5 young men, captured from their clavicles to jaws (front and left side views), without showing facial features. The videos included laryngeal lifting action and swallowing sound at 1500 ms. Ten different swallowing videos were randomly presented as cue materials, with each video displayed twice during the 20 trials. In this study, the 40 trials in NISI task, while the 20 trials in AISI task.

### Data preprocessing and source reconstruction

The MRI data were processed using BrianSuite18a^29^. The whole brain was divided into 130 ROIs according to the USCBrain-BrainSuite-2017 atlas^30^. The ROIs were then mapped onto the Montreal Neurological Institute coordinate system to create the anatomical brain model. The anatomical modeling files and the original MEG data were imported into Brainstorm^31^ for further analysis. The MEG data underwent preprocessing steps, including band-pass filtering with a range of 1 Hz to 40 Hz, removal of bad channels, and elimination of artifacts caused by eye movements (EOG), cardiac activity (ECG), and muscle activity (EMG). Signal space projection (SSP) and independent component analysis (ICA) techniques were employed to detect and remove these artifacts. Source reconstruction was performed on broadband MEG data first; the reconstructed source files were then filtered into alpha (8 ~ 13 Hz) and beta (14 ~ 29 Hz) frequency bands for subsequent ERSP and connectivity analyses.

### Construction process of averaged functional networks

Two types of averaged functional networks were constructed to serve distinct analytical purposes: intra-subject averaged files for correlation analysis, and inter-subject averaged networks for visualization of global node distribution.

For intra-subject averaging (used in amplitude correlation analysis), each of the 14 subjects contributed task-specific averaged source files: 40 valid trials for non-induced swallowing imagery (NISI) and 20 valid trials for action observation-induced swallowing imagery (AISI) were averaged per subject, yielding 14 intra-subject averaged files for NISI and 14 for AISI. These files preserved individual variability and were used to compute correlations between regional activity (ERSPs) and network parameters, followed by group-level comparisons.

For inter-subject averaging (used in global node distribution visualization), the 14 intra-subject averaged files for each task were further averaged across subjects to generate two group-level files (one for NISI, one for AISI). Using these, functional networks were constructed separately for the alpha (8–13 Hz) and beta (14–29 Hz) bands via corrected imaginary phase locking values (ciPLVs) between 130 ROIs, resulting in four inter-subject averaged networks (NISI-alpha, NISI-beta, AISI-alpha, AISI-beta) to visualize spatial patterns of node types.

### Activity of ROIs

The reconstructed source files were utilized for time–frequency analysis of event-related spectral perturbations (ERSPs) using Brainstorm software^32^. The ERSPs represented the power spectral density of non-phase-locked signals during motor imagery. In this study, a baseline period of − 5 s to − 3 s was selected, and a task period from 0 to 10 s was selected for analysis. The power during the task period was denoted as Et, and the power during the baseline period was denoted as Eb. The ERSPs were calculated as (Et − Eb)/Eb. ERSPs greater than 0 indicated event-related synchronization (ERS), while ERSPs less than 0 indicated desynchronization (ERD)^33–36^. The ERSPs of the two tasks in the α (8 ~ 13 Hz) and β (14 ~ 29 Hz) frequency bands were computed, and the ERSPs were utilized as the activity index of the ROIs.

### Graph modeling

Based on graph theory, we considered the 130 ROIs as nodes, and the synchronous activity relationship of ROIs as edges (i.e., connected lines). Phase locking values (PLVs) was commonly used to measure the functional connectivity between ROIs^37–40^. However, the estimation of synchronization is inevitably influenced by challenges associated with volume conduction and source leakage, thereby necessitating the application of advanced correction techniques. Bruña et al.^41^ have emphasized the remarkable sensitivity of the corrected imaginary phase locking values (ciPLVs) to volume conductivity, which allows for a more precise representation of the genuine synchronicity. Consequently, in this study, the Brainstorm software^31^ was utilized to compute the ciPLVs between ROIs and establish an undirected weighted brain functional network, with these ciPLVs employed as the connection weights. Edge weights were derived from ciPLV values, which range from 0 to 1 and are positively correlated with the synchronization and connectivity strength between channels—values approaching 1 signify stronger inter-channel interactions, whereas lower values correspond to weaker connections. In order to filter spurious connections, we referred to the method proposed by Gonuguntla et al.^42^. The 1.5 times of the averaged ciPLVs of the two tasks were set as the threshold. The edges with weights lower than the threshold would be removed from the network:

$$Threshold = 1.5*\frac{{\left\{ {\left[ {ciPLVs_{{}}^{AISI} } \right] + \left[ {ciPLVs_{{}}^{NISI} } \right]} \right\}}}{2}$$

The mean ciPLVs during AISI or NISI tasks (0 s ~ 10 s) were denoted as $\left[ {ciPLVs_{{}}^{AISI} } \right]$ and $\left[ {ciPLVs_{{}}^{NISI} } \right]$. According to the method above, we constructed undirected weighted networks for each subject in alpha and beta bands. Additionally, we averaged the sourced files of all subjects and created averaged networks for each task in the alpha and beta bands. Moreover, 130 ROIs were classified as eight functional systems according to the functional scheme^43^: ① frontoparietal system; ② attention system; ③ motor and somatosensory system; ④ cinguloopercular system; ⑤ visual system; ⑥ medial default mode system; ⑦ ventral temporal association system; ⑧ auditory system.

### Network parameters of ROIs

We used igraph 1.2.6 package^44^ and brainGraph 3.0.0 package^45^ in the R software to calculate the network parameters for each functional brain network of each subject. Seven parameters were calculated for each node in the network. Node *i* was taken as the example, where *i* can be any node in the network. The nodes directly connected to the node *i* were called adjacent nodes of *i*. The concept and meaning of these parameters were described as follows:

Seven network parameters were calculated as follows. The first five were weighted parameters, and the last two were unweighted ones. The *weighted degree* was computed based on the number of adjacent nodes and the edge weights associated with node *i*. To evaluate the proximity between node *i* and other nodes in the network, the *weighted closeness centrality* measure is employed. A higher value indicates an increased likelihood of information dissemination from node *i* to any other node in the network. The *weighted betweenness centrality* quantifies whether a node lies on the shortest path connecting any two nodes, thereby assessing its criticality in information transmission. The *weighted eigenvector centrality* not only determines the extent of connectivity for node *i* but also evaluates the connectivity of its adjacent nodes. Thus, nodes with high eigenvector values have a significant influence on the overall network and serve as indicators for detecting network hubs^22,46^. The *weighted transitivity* measures the connectivity between the adjacent nodes of node *i*. *K-coreness* is an unweighted measure that divides the entire network into subnetworks. For instance, if the k-coreness of node *i* is 4, it signifies that each node within the subnetwork has an unweighted degree greater than or equal to 4. *Laplacian centrality* characterizes the impact of node removal on the network structure. It quantifies the extent to which the network’s structural properties would be affected if node *i* were removed.

### Node type division

In the averaged network, the index for measuring activity was the value of ERSPs. ROIs exhibiting activity greater than the mean plus standard deviation were classified as hotspots, while the remaining nodes were designated as cold spots^22,47^. Furthermore, the weighted eigenvector of each node was utilized to differentiate between hubs and non-hubs, with nodes possessing top-level weighted eigenvector centrality considered as hubs. However, the determination of hubs lacks a consistent method, thus we referred to previous studies to maintain consistency between the number of hubs and hotspots^22^.

Ultimately, based on activity and connectivity, the 130 ROIs were categorized into four types: hot hubs (HHs) characterized by strong activity and strong connectivity, cold hubs (CHs) exhibiting weak activity but strong connectivity, non-hub hotspots (nh-HSs) showing strong activity but weak connectivity, and non-hub cold spots (nh-CHs) demonstrating weak activity and weak connectivity. Additionally, the subnetwork comprised of cold hubs was designated as the dark functional network^22^.

### Statistical analysis

In the present study, the correlation analysis between regional activity (ERSPs) and network parameters was conducted as an exploratory analysis rather than inferential hypothesis testing. Our primary goal was to characterize the potential association patterns between activity and connectivity across 130 ROIs, rather than performing multiple independent statistical tests to verify pre-specified hypotheses.

Statistical significance of each correlation was evaluated using a two-tailed t-test to assess whether the observed correlation differed from zero, with results reported as Pearson correlation coefficients, 95% confidence intervals (95%CI) of ERSPs and seven network parameters were calculated by psych 2.1.3 package^48^ in the R software (*p* < 0.05). In this study, a significant positive correlation between activity and connectivity, as measured by the Pearson coefficient, was considered a positive activity-connectivity coupling relationship. Conversely, a significant negative correlation between activity and connectivity was considered a negative activity-connectivity coupling relationship. If there was no significant correlation, the activity and connectivity were considered uncoupled. Network visualization was performed using Pajek5 13^46^ and VOSviewer 1.6.13^49^.

## Results

### Analysis of hot hubs, non-hub hotspots and cold hubs

**Fig. 3:**
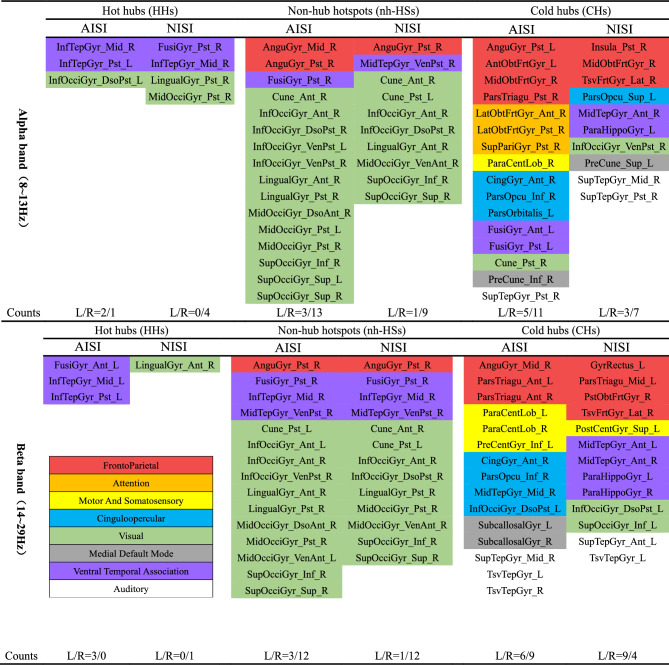
Analysis of hot hubs, non-hub hotspots and cold hubs. Figure 3 illustrates the distribution of hot hubs, non-hub hotspots, and cold hubs across the bilateral cerebral hemispheres in the alpha and beta frequency bands for the two tasks (NISI and AISI), and displays the number of activated brain regions in the left and right hemispheres (Counts L/R). Colors for functional modules: red: frontoparietal system; orange: attention system; yellow: motor and somatosensory system; blue: cinguloopercular system; green: visual system; grey: medial default mode system; purple: ventral temporal association system; white: auditory system. NISI: non-induced swallowing imagery; AISI: action observation-induced swallowing imagery.

Figure 3 depicts the distribution of hot hubs, non-hub hotspots, and cold hubs across the bilateral brain hemispheres within the alpha and beta frequency bands (**Attachment 2** for the full name of the abbreviation). The figure provides a count of these regions in both the left and right hemispheres, allowing for a comprehensive understanding of their spatial distribution. Additionally, the color-coded visualization distinguishes eight functional systems. In the alpha band, both tasks exhibit similar patterns of hot hubs, predominantly involving the ventral temporal association system and the visual system. Regarding non-hub hotspots, both tasks activate identical functional systems, yet the AISI task invoke more ROIs within the visual system. In terms of CHs, AISI task demonstrates a higher number of invoked ROIs associated with the cinguloopercular system, the motor and somatosensory system, as well as the attention system. Moreover, the AISI task showcases increased the number of the non-hub hotspots and CHs in both brain hemispheres when compared to the NISI task.

Within the beta band, the AISI exhibits more invoking of hot hubs, involving the ventral temporal association system. Concerning non-hub hotspots, both tasks activate the same functional systems, but the AISI task demonstrates invoking more ROIs within the visual system. Notably, there are distinct variations in the activated functional systems between the two tasks regarding CHs. The AISI task exhibits heightened activation of ROIs associated with the motor and somatosensory system, the cinguloopercular system and the medial default mode system. In contrast, the NISI task predominantly activates the ventral temporal association system and the visual system. Additionally, the AISI task exhibits more activation of non-hub hotspots in both brain hemispheres compared to the NISI task.

### Correlation analysis

**Fig. 4:**
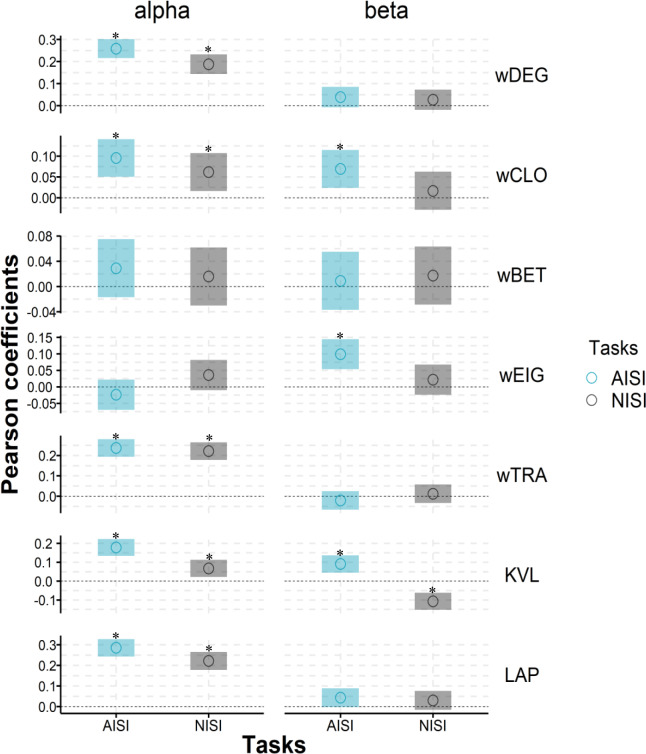
Correlation between amplitude and network parameters.The figure shows the Pearson coefficients (circle) and its 95% confidence intervals (transparent color bar) of ERSPs and seven module-independent parameters of both tasks in alpha and beta bands. The * represents that the correlation coefficient is significant (*p* < 0.05). The Pearson coefficient is equal to 0, which is displayed with a horizontal dotted line. wDEG: weighted degree centrality; wBET: weighted betweenness centrality; wEIG: weighted eigenvector centrality; wTRA: weighted transitivity; KVL: k-value of coreness; LAP: Laplacian centrality. NISI: non-induced swallowing imagery, AISI: action observation-induced swallowing imagery.

Figure 4 presents the Pearson correlation coefficients between activity (ERSPs) and network parameters. Significant differences at the 95% confidence level are denoted by “*” (*p* < 0.05). Despite the presence of a coupling relationship between activity and connectivity in both tasks, the correlation coefficients are low, suggesting weak associations^50^. This indicates that activity and connectivity of ROIs represent distinct evaluative systems, and a combined analysis can yield insights into more valuable brain regions. In the alpha band, both tasks demonstrate similar coupling patterns. Weighted degree, weighted closeness centrality, weighted transitivity, k-coreness, and Laplacian centrality exhibit positive activity-connectivity coupling in both tasks, while the remaining parameters demonstrate decoupling relationships. However, in the beta band, the two tasks exhibit distinct coupling patterns. During the AISI task, weighted closeness centrality, weighted eigenvector centrality, and k-coreness show positive activity-connectivity coupling, while the other parameters exhibit decoupling relationships. In the NISI task, only k-coreness exhibits a negative coupling relationship, while the other parameters show decoupling relationships.

### Visualization of brain functional networks

**Fig. 5:**
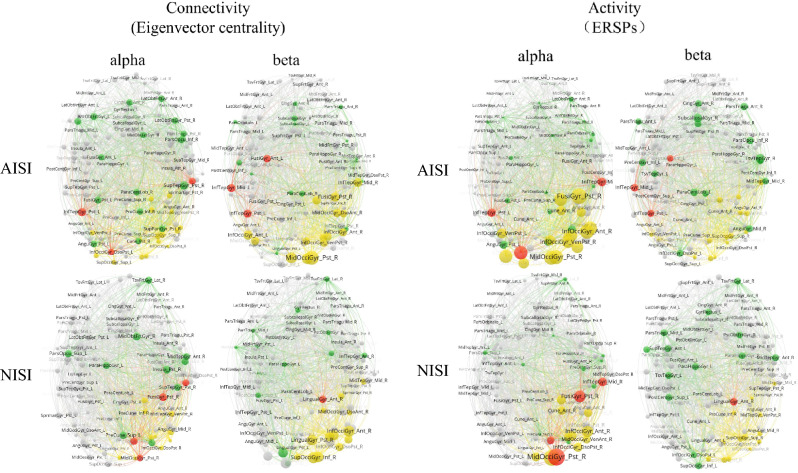
Two tasks of brain functional network visualization. Red: hot hubs Green: cold hubs, Yellow: non-hub hotspots, Gray: non-hub cold spots. The left panel shows the layout of connectivity of both tasks in alpha and beta bands. The node size is directly proportional to the eigenvector centrality. The activity layouts of both tasks in alpha and beta bands are shown on the right panel, and the node size is directly proportional to the absolute value of ERSPs. The perspective of all pictures of brain networks are top-down. NISI: non-induced swallowing imagery, AISI: action observation-induced swallowing imagery.

Figure 5 depicts the visualization of averaged network data and the classification of nodes into four distinct types. Specifically, node size in the left panel is positively correlated with the functional connectivity of nodes, while that in the right panel is positively correlated with node activity strength. Each network exhibited a limited number of hot hubs (depicted in red), primarily distributed in the occipital and temporal lobes for both tasks. In the alpha and beta frequency bands, non-hub hotspots (depicted in yellow) of the NISI task mainly localized to the right occipital lobe, while the AISI task exhibited a broader distribution, encompassing both occipital lobes. Concerning cold hubs (depicted in green), in both frequency bands, both tasks predominantly activated cold hubs in the bilateral frontal and limbic systems. Notably, the AISI task demonstrated a higher number of invoked cold hubs, specifically in the alpha band.

## Discussion

In the present study, we focused on the activity and functional connectivity of ROIs. We explored the brain processing mechanisms during non-induced and action observation-induced swallowing imagery from the perspective of the activity-connectivity relationship of ROIs. Our study revealed three main findings: (1) High-activity ROIs are not synonymous with high-connectivity ROIs, and such a dissociation underscores the functional diversity of hub regions—specifically, certain hubs can fulfill their connectivity functions independent of high levels of neural activation. (2) In AISI, we observed increased activation of non-hub hotspots within the visual system, along with an augmented presence of cold hubs in the motor and somatosensory, frontoparietal, and cinguloopercular systems. (3) AISI enhanced the recruitment patterns of the hotspot network and the dark functional network to be more focused on swallowing-related regions and increased functional connectivity in both hemispheres.

### Activity-connectivity coupling relationship

Studies investigating crucial nodes in the cerebral cortex during task-related processes often utilize neuroimaging techniques to examine highly activated brain regions, known as “hotspots,” and intervene accordingly. With the development of brain functional connectivity analysis, researchers have started using functional connectivity analysis as a complementary strategy to investigate the activation levels in brain regions. This approach helps identify highly connected nodes during task-related processes, which is crucial for understanding brain signal processing. In this study, we employed both magnetoencephalography (MEG) and functional connectivity analysis to explore cortical invocation patterns in healthy young individuals during the execution of a swallowing imagery task under different conditions.

In the alpha frequency band, both tasks demonstrated similar activity-connectivity coupling relationships, with all significant correlations displaying a positive coupling pattern. This suggests that highly active ROIs tend to exhibit high connectivity (as measured by weighted degree) and possess stable local structures (as measured by weighted transitivity). Additionally, highly active ROIs were more likely to be located in the core modules (as indicated by k-coreness) and occupy critical positions (as indicated by Laplacian centrality), facilitating efficient information transmission to any node within the network (as indicated by weighted closeness centrality). Although no significant differences were observed in the correlation coefficients between the two tasks, the AISI task exhibited higher positive coupling coefficients compared to the NISI task. This indicates that AISI enhanced the connectivity of highly active ROIs to some extent.

In the beta frequency band, only the AISI task displayed positive coupling relationships in weighted closeness centrality, weighted eigenvector centrality, and k-coreness. In the NISI task, both weighted closeness centrality and weighted eigenvector centrality showed decoupling relationships, while k-coreness exhibited a negative coupling relationship. This further suggests that AISI led to denser connections among highly active ROIs (as measured by weighted eigenvector centrality) and facilitated more efficient information transmission (as measured by weighted closeness centrality). However, the relatively low correlation coefficients (correlation coefficient < 0.3) between activity and functional connectivity indicate a limited enhancement of connectivity by AISI. The decoupling of weighted betweenness centrality also implies that information transmission can occur through multiple ROIs. Despite the relatively high connectivity of highly active ROIs, they do not necessarily act as key messengers in the process of brain network information transmission. In this study, weighted eigenvector centrality was employed as an indicator to determine whether an ROI is a hub. However, it only showed decoupling or weak positive coupling relationships. This indicates that hot spots and hubs may not completely overlap. Therefore, traditional intervention methods that solely target stimulation hotspots may face challenges in influencing the entire brain functional network^51^, as regulating cold hubs that govern information transmission within the network also plays a critical role during tasks.

### Invoking differences of hot hubs, non-hub hotspots and cold hubs

In the alpha frequency band, both tasks demonstrated a limited number of activated hot hubs (Fig. 4). Previous investigations have documented the activation of these brain regions, such as the temporal gyrus, insula, cingulate gyrus, and occipital cortex, during swallowing tasks^8,52,53^. However, different from the execution of swallowing tasks, the somatosensory and motor cortex, which are highly relevant to swallowing actions, did not exhibit hot hubs invocation in either the alpha or beta frequency bands for both tasks. Instead, the activated hot hubs in both tasks were primarily associated with visual functions, including the lingual gyrus, fusiform gyrus, middle occipital gyrus, and inferior occipital gyrus. Participants’ self-ratings of swallowing imagery, assessed using the KVIQ-10 scale, indicated a significant higher score for visual swallowing imagery over kinesthetic swallowing imagery (Table 1). While we instructed the participants to use kinesthetic imagery strategies as much as possible, self-assessment and cortical activation results suggest that visual imagery strategies were more readily employed by the participants. Consequently, participants may have a tendency to imagine swallowing scenes, leading to the activation of hot hubs in the visual cortex. Furthermore, it is important to note that swallowing is accompanied by auditory experiences, specifically the sounds associated with the swallowing process. Participants may in vividly imagining the sound of swallowing, which can result in increased activity in the temporal lobe.

The involvement of attention-related ROIs (localized to parietal and frontal cortices) as nonhub hotspots in our alpha-band results gains critical mechanistic support from recent MEG work. Bagherzadeh et al.^54^ demonstrated a causal role of parietal alpha oscillations in spatial attention control using MEG neurofeedback: upregulating alpha lateralization in parietal cortex suppressed irrelevant hemispheric activity to enhance sensory processing of task-relevant stimuli, with effects persisting in subsequent attention tasks (e.g., Posner paradigm). This aligns with our observation that attention-related ROIs were concentrated as nonhub hotspots in the alpha band—suggesting that alpha oscillations in swallowing imagery similarly support attentional selection by biasing neural resources toward task-critical regions (e.g., visual and swallowing-related ROIs). Additionally, De Vries et al.^55^ showed that source-reconstructed alpha oscillations enable successful decoding of behavioral task states; their MEG study decoded attended auditory streams from alpha activity in 360 ROIs, with accuracy correlating directly with task performance. In our study, alpha-band weighted closeness centrality (a key connectivity parameter for information transmission) showed stronger positive coupling with ERSP amplitude in AISI than NISI—implying that alpha-mediated connectivity patterns in AISI may better encode task-specific imagery states, echoing De Vries et al.’s finding that alpha oscillations support task-relevant decoding.

Regarding cold hubs, in the alpha frequency band, the AISI task exhibited additional activation of ROIs from the attention system, motor and somatosensory system, and the cinguloopercular system compared to the NISI task (Fig. 4). Similarly, in the beta frequency band, the AISI task exhibited increased invocation of ROIs from the motor and somatosensory system and the cinguloopercular system. These findings align with previous studies. Babaei et al.^14^ reported that the motor and somatosensory cortex exhibited high connectivity in healthy young individuals during the execution of swallowing actions. Martin et al.^8^ reported that swallowing tasks not only require activation of the motor and somatosensory cortex but also engagement of cognitive and attentional brain regions. Compared to swallowing execution, swallowing imagery tasks are more complex and require greater cognitive involvement^36^. Additionally, the invocation of the cinguloopercular system facilitates the integration of somatosensory motor information^56^. AISI enables participants to be more focused on the execution of the task while enhancing their processing capabilities for motor and somatosensory information.

### Separation pattern of hotspots network and dark functional network

The presence of the “dark matter” of the brain has demonstrated that energy is not only for increase neuronal excitability but also for information transmission between neurons^57–59^. Cold hubs represent unique brain regions characterized by low energy but high connectivity. In our study, we refer to the sub-network consisting of these cold hubs as the “dark functional network”^22^. The presence of these regions helps to minimize overall energy consumption while maintaining them in a “standby” state. When a specific task requires the activation of a distant brain region, the dark functional network can promptly respond and transmit relevant information to facilitate the desired location’s energy state. Dark functioning network offers a new insight to investigate the activity and connectivity of ROIs.

Our approach to constructing functional networks using corrected imaginary phase locking values (ciPLVs)—a metric capturing ROI-level synchrony—aligns with recent MEG work by Mantegna et al.^60^, who demonstrated that spatial covariance patterns across ROIs decode imagined stimulus categories. Mantegna et al. used covariance matrices of alpha/beta-band activity to classify imagined visual objects (faces vs. places), revealing category-specific connectivity networks (e.g., occipital-temporal covariance for object imagery). In our study, AISI (which includes visual observation) exhibited stronger occipital-parietal alpha covariance than NISI—mirroring Mantegna et al.’s finding that external cues (e.g., action observation in our case) shape imagery-related connectivity covariance. Notably, Mantegna et al. emphasized that connectivity covariance outperforms single-ROI activity for decoding imagery content—supporting our focus on activity-connectivity coupling rather than isolated node metrics.

Wang et al.^61^ utilizing resting-state fMRI and swallowing behavior assessments in patients with mild cognitive impairment and swallowing difficulties, as well as healthy controls. By utilizing regional homogeneity (ReHo) to assess functional connectivity between brain regions, they found a significant correlation between ReHo values and swallowing performance. Compared to the healthy control group, patients exhibited poorer swallowing performance, alongside significantly lower ReHo values in the bilateral inferior occipital lobes (IOLs) and the left anterior frontal lobe. The bilateral IOLs and left anterior frontal lobe are associated with swallowing function and cognitive levels. In the present study, we observed similar results, with the prefrontal cortex and occipital cortex activated as hot hubs or cold hubs, exhibiting high connectivity in both tasks. This suggests that swallowing imagery training holds promising potential in enhancing swallowing function and performance.

In this study, both tasks exhibited a clear segregation between the hotspots network (composed of hot hubs and non-hub hotspots) and the dark functional network in the alpha and beta frequency bands, with little overlap observed between highly active and highly connected ROIs. The hotspots network primarily involved the occipital and temporal cortices, while the dark functional network predominantly encompassed the frontal, parietal, and limbic systems. This distribution of sub-networks suggests that the hotspot functional network may be associated with the utilization of visual imagery strategies, while the dark functional network may play a role in the planning of complex mental imagery and the processing and integration of information from cognitive and somatosensory motor systems. Previous research has proposed that high-activity hotspots are related to task performance and that highly connected dark functional networks are crucial for signal processing^22^, which aligns with our findings.The identification of this segregation pattern between the hotspot network and the dark network in healthy individuals provides valuable insights for the treatment of individuals with brain injuries. For example, in cases where the connectivity of the dark functional network is impaired in patients, the use of synchronized small stimuli in multiple cold hubs areas to intervene the brain network may facilitate the improvement of swallowing disorders^62^.

In contrast to limb movements, which typically exhibit lateralization^63,64^, swallowing is a non-lateralized and midline action. During swallowing tasks, various brain regions involved in swallowing exhibit activation and increased connectivity in both cerebral hemispheres, without significant interhemispheric differences^6–9,14,15^. In terms of activity, the NISI task primarily exhibited high activity ROIs (hot hubs and non-hub hotspots) in the right occipital lobe in both frequency bands, whereas the AISI task increased the number of high activity ROIs and expanded their distribution to include the bilateral occipital lobes. Regarding connectivity, the AISI task exhibited enhanced connectivity of the ROIs in the frontal lobes, resulting in an increased number of cold hubs and the generalization of the dark functional network to both hemispheres, particularly in the alpha frequency band. AISI has demonstrated promising results in enhancing the activity and connectivity of brain regions in both hemispheres, leading to increased efficiency in information transmission and resource utilization within the brain’s functional network. The improvement in both activity and connectivity indicates that action observation has a priming and enhancing effect on swallowing imagery^65^. This intervention shows significant potential for optimizing the recruitment patterns within the overall brain functional network, resulting in a more efficient and coordinated network for cognitive and motor functions. These findings highlight the significance of action observation combined with swallowing imagery as a potential therapeutic intervention for enhancing brain function and improving neural communication.

Overall, our study contributes to the understanding of the brain’s functional network during swallowing imagery tasks and highlights the potential of action observation-induced swallowing imagery as a valuable tool for modulating brain activity and connectivity in relation to swallowing imagery.

## Limitations and future direction

This study has several limitations that should be acknowledged. Firstly, our analysis focused solely on the activity-connectivity coupling relationships within the alpha and beta frequency bands. This choice was based on the highly band-specific nature of event-related spectral perturbations (ERSPs), which are more prominently observed in the alpha and beta bands^33^. Future investigations should explore activity-connectivity relationships across a wider range of frequency bands to obtain a more comprehensive understanding.

Secondly, the sample size in our study was small, consisting solely of healthy young adults. Although we determined the sample size based on previous studies^22,36,66^, the generalizability of our findings to other populations may be limited. Future studies should aim to include larger and more diverse samples, including older adults and stroke patients with impaired swallowing function. Additionally, incorporating non-invasive brain stimulation and brain imaging techniques in clinical practice would allow for the administration of different stimulations to hot hubs and cold hubs, facilitating a deeper understanding of changes in brain network recruitment patterns and their impact on swallowing function in patient populations.

Thirdly, there were limitations in the connectivity modeling of magnetoencephalography (MEG) data. The cortical activity recorded by sensors required cortical source reconstruction, but signal leakage from the same sensor into adjacent spaces during reconstruction could lead to spurious cortical connections. Previous studies have suggested that the weighted minimum-norm estimation (WMNE) method provides more accurate source estimates compared to the beamformer method, thus reducing artifactual connections caused by signal leakage^47,67^. Additionally, we employed the corrected imaginary phase locking value (ciPLV), a recently introduced method, to estimate the synchrony between ROIs. Compared to traditional methods such as the imaginary part of coherency (ImC) and the phase lag index (PLI), ciPLV offers advantages such as reduced susceptibility to phase delays and lower sensitivity to volume conduction. While current techniques cannot completely eliminate spurious connections, addressing this challenge remains an ongoing effort.

Despite these limitations, our study offers preliminary support for the exploration of brain functional network recruitment patterns through the lens of activity-connectivity correlation. The stability of brain network recruitment patterns observed in young healthy adults provides a foundation for future investigations involving larger and more diverse samples. Furthermore, studies involving aging individuals and stroke patients, combined with non-invasive brain stimulation and imaging techniques, can shed light on changes in brain network recruitment patterns and their relationship to swallowing function, ultimately contributing to our understanding of the underlying brain mechanisms.

In summary, while acknowledging these limitations, this study provides valuable evidence for the utility of activity-connectivity-based research methods and opens avenues for further exploration using alternative synchrony estimation approaches, such as symmetric orthogonalization procedures^68^, full source-space interaction mapping^69^, and the construction of directed networks using phase transfer entropy to investigate information flow direction in brain networks^70,71^.

## Conclusion

In conclusion, this study investigated the recruitment patterns of functional brain networks during swallowing imagery tasks from an activity-connectivity perspective. The results revealed that high-activity regions did not necessarily exhibit high connectivity, highlighting the complex nature of brain network organization. Action observation-induced swallowing imagery techniques showed promising effects in enhancing the recruitment of non-hub hotspots and cold hubs leading to optimizing information processing efficiency within the brain network. The identification of distinct patterns in the hotspots network and the dark functional network provides valuable insights into the understanding of brain network dynamics during swallowing tasks. Moreover, correlation analysis between activity and connectivity and the detection of dark functional networks offered novel approaches for studying brain network recruitment patterns. These findings contribute to the field of brain research and have implications for the selection of therapeutic targets in noninvasive brain stimulation for swallowing disorders. Future studies can further explore the underlying mechanisms of activity-connectivity coupling and investigate the potential clinical applications of these findings in improving other function in patient populations.

The full names of the brain regions mentioned in this paper are provided in Appendix 2.

## Supplementary Information

Supplementary Information.

## Data Availability

The datasets used and/or analysed during the current study are available from the corresponding author on reasonable request.

## Abbreviations

AISI: Action observation-induced swallowing imagery
NISI: Non-induced swallowing imagery
MEG: Magnetoencephalography
ROIs: Regions of interest
ciPLVs: Corrected imaginary phase locking values
ERSPs: Event-related spectral perturbations
ERD: Event-related desynchronization
ERS: Event-related synchronization
KVIQ-10: Kinesthetic and visual imagery questionnaire-10
MRI: Magnetic resonance imaging
wDEG: Weighted degree
wCLO: Weighted closeness centrality
wBET: Weighted betweenness centrality
wEIG: Weighted eigenvector centrality
wTRA: Weighted transitivity
KVL: K-coreness
LAP: Laplacian centrality
