# Supplementary material for: Recruitment patterns of hothubs and dark functional networks correlating activity and connectivity with magnetoencephalography
Source: Sci Rep. 2026 Jan 16;16:4665. doi: 10.1038/s41598-025-34860-0 (PMC12868798; doi:10.1038/s41598-025-34860-0)
Supplement: Supplementary file 1 — Supplementary Information. [file 41598_2025_34860_MOESM1_ESM.zip › Appendix files/Appendix 1.Experimental paradigm and data preprocessing.docx]

**Data acquisition**

MEG data was recorded using a whole-head CTF-275 channel system in a magnetically shielded room (VSM Medical Technologies, Canada), with a sampling rate of 1200 Hz. Before the MEG scanning, electroencephalogram (EEG) leads were placed bilaterally above and below the orbitals, outer canthus, wrist, dorsum of the hand, bilateral submental and infrahyoid muscles to capture and eliminate undesired activity of eyes, heart, and muscles. In addition, marking the nasion and bilateral pre-auricular points and attaching the coils to locating the head position. Data with head displacements exceeding 0.5 cm were excluded from the analysis. During the MEG scanning, participants wore plastic earphones and were instructed to lie flat on the scanning bed. Visual stimuli were presented on a screen positioned 40 cm in front of the subject, and the image and sound were adjusted for clarity and comfort. The task stimuli, including pictures or videos, were presented using BrainX software (Cincinnati Children's Hospital Magnetoencephalography Center, USA) (Dinga *et al.* 2018, Xiang *et al.* 2001). Participants were instructed to minimize limb and head movements and to avoid blinking or swallowing actions as much as possible. Following the MEG recording, T1-weighted images were acquired using a 1.5T MRI system (GE, USA) to obtain individual anatomical brain data (TR: 33 ms; TE: 9 ms; recording matrix: 256 × 256 pixels; excitation: 1; the field of view: 240 mm; and slice thickness: 1.4 mm). To ensure consistent head positions between the MEG and MRI scans, vitamin E pellets were placed at the same positions as the marked points (nasion and bilateral pre-auricular points).

Refernce

Dinga, S., Wu, D., Huang, S., Wu, C., Wang, X., Shi, J., Hu, Y., Liang, C., Zhang, F., Lu, M., Leiken, K., and Xiang, J., 2018, Neuromagnetic correlates of audiovisual word processing in the developing brain. *International Journal of Psychophysiology*, **128**, 7–21. https://doi.org/10.1016/j.ijpsycho.2018.03.016.

Joshi, A.A., Choi, S., Sonkar, G., Chong, M., Gonzalez-Martinez, J., Nair, D., Shattuck, D.W., Damasio, H., and Leahy, R.M., 2017, A whole brain atlas with sub-parcellation of cortical gyri using resting fMRI. In M.A. Styner and E.D. Angelini (eds) Presented at the SPIE Medical Imaging, ((Orlando, Florida, United States)), p. 101330O.

Shattuck, D.W. and Leahy, R.M., 2002, BrainSuite: an automated cortical surface identification tool. *Medical Image Analysis*, **6**, 129–142. https://doi.org/10.1016/s1361-8415(02)00054-3.

Tadel, F., Bock, E., Niso, G., Mosher, J.C., Cousineau, M., Pantazis, D., Leahy, R.M., and Baillet, S., 2019, MEG/EEG Group Analysis With Brainstorm. *Frontiers in Neuroscience*, **13**, 76. https://doi.org/10.3389/fnins.2019.00076.

Xiang, J., Wilson, D., Otsubo, H., Ishii, R., and Chuang, S., 2001, Neuromagnetic spectral distribution of implicit processing of words. *Neuroreport*, **12**, 3923–3927. https://doi.org/10.1097/00001756-200112210-00014.
