# Supplementary material for: Recruitment patterns of hothubs and dark functional networks correlating activity and connectivity with magnetoencephalography
Source: Sci Rep. 2026 Jan 16;16:4665. doi: 10.1038/s41598-025-34860-0 (PMC12868798; doi:10.1038/s41598-025-34860-0)
Supplement: Supplementary file 1 — Supplementary Information. [file 41598_2025_34860_MOESM1_ESM.zip › Appendix files/Appendix 2_abbreviation.docx]

The abbreviation of brain regions corresponds to the full name in the text.

| **Abbreviation** | | **Full name** | **Abbreviation** | | **Full name** |
| --- | --- | --- | --- | --- | --- |
| AnguGyr_Mid_L | angular gyrus_middle L | | | AnguGyr_Pst_R | angular gyrus_posterior R |
| AntObtFrtGyr_L | anterior orbito-frontal gyrus L | | | AntObtFrtGyr_R | anterior orbito-frontal gyrus R |
| GyrRectus_L | gyrus rectus L | | | GyrRectus_R | gyrus rectus R |
| Insula_Ant_L | insula_anterior L | | | Insula_Pst_R | insula_posterior R |
| Insula_Pst_L | insula_posterior L | | | MidFrtGyr_Pst_R | middle frontal gyrus_posterior R |
| ParsTriagu_Pst_L | pars triangularis_posterior L | | | MidObtFrtGyr_R | middle orbito-frontal gyrus R |
| PstObtFrtGyr_L | posterior orbito-frontal gyrus L | | | PstObtFrtGyr_R | posterior orbito-frontal gyrus R |
| TsvFrtGyr_Lat_L | transverse frontal gyrus_lateral L | | | TsvFrtGyr_Msl_R | transverse frontal gyrus_mesial R |
| LatObtFrtGyr_Ant_L | lateral orbitofrontal gyrus_anterior L | | | LatObtFrtGyr_Pst_R | lateral orbitofrontal gyrus_posterior R |
| SupPariGyr_Pst_L | superior parietal gyrus_posterior L | | | ParaCentLob_R | paracentral lobule R |
| ParaCentLob_L | paracentral lobule L | | | PostCentGyr_Sup_R | postcentral gyrus_superior R |
| PostCentGyr_Sup_L | postcentral gyrus_superior L | | | PreCentGyr_Sup_R | precentral gyrus_superior R |
| PreCentGyr_Inf_L | precentral gyrus_inferior L | | | Cune_Pst_R | cuneus_posterior R |
| PreCentGyr_Sup_L | precentral gyrus_superior L | | | InfOcciGyr_Ant_R | inferior occipital gyrus_anterior R |
| MidFrtGyr_Ant_L | middle frontal gyrus_anterior L | | | LingualGyr_Pst_R | lingual gyrus_posterior R |
| ParsOrbitalis_L | pars orbitalis L | | | MidOcciGyr_Pst_R | middle occipital gyrus_posterior R |
| SprmarGyr_Ant_L | supramarginal gyrus_anterior L | | | SupOcciGyr_Sup_R | superior occipital gyrus_superior R |
| LingualGyr_Pst_L | lingual gyrus_posterior L | | | SupFrtGyr_Ant_R | superior frontal gyrus_anterior R |
| CingGyr_Pst_L | cingulate gyrus_posterior L | | | SupFrtGyr_Pst_R | superior frontal gyrus_posterior R |
| PreCune_Inf_L | precuneus_inferior L | | | MidTepGyr_DsoPst_R | middle temporal gyrus_dorsoposterior R |
| PreCune_Sup_L | precuneus_superior L | | | MidTepGyr_VenPst_R | middle temporal gyrus_ventroposterior R |
| SubcallosalGyr_L | subcallosal gyrus L | | | ParaHippoGyr_R | parahippocampal gyrus R |
| FusiGyr_Ant_L | fusiform gyrus_anterior L | | | TsvTepGyr_R | transverse temporal gyrus R |
| FusiGyr_Pst_L | fusiform gyrus_posterior L | | | AnguGyr_Ant_R | angular gyrus_anterior R |
| InfTepGyr_Mid_L | inferior temporal gyrus_middle L | | | ParsTriagu_Mid_R | pars triangularis_middle R |
| InfTepGyr_Pst_L | inferior temporal gyrus_posterior L | | | TsvFrtGyr_Lat_R | transverse frontal gyrus_lateral R |
| MidTepGyr_DsoPst_L | middle temporal gyrus_dorsoposterior L | | | LatObtFrtGyr_Ant_R | lateral orbitofrontal gyrus_anterior R |
| MidTepGyr_Mid_L | middle temporal gyrus_middle L | | | SupPariGyr_Pst_R | superior parietal gyrus_posterior R |
| MidTepGyr_VenPst_L | middle temporal gyrus_ventroposterior L | | | ParsOpcu_Inf_R | pars opercularis_inferior R |
| TepPole_L | temporal pole L | | | PreCune_Inf_R | precuneus_inferior R |
| Cune_Ant_L | cuneus_anterior L | | | FusiGyr_Ant_R | fusiform gyrus_anterior R |
| MidOcciGyr_DsoAnt_L | middle occipital gyrus_dorsoanterior L | | | MidTepGyr_Ant_R | middle temporal gyrus_anterior R |
| MidOcciGyr_VenAnt_L | middle occipital gyrus_ventroanterior L | | | SupTepGyr_Ant_R | superior temporal gyrus_anterior R |
| SupFrtGyr_Ant_L | superior frontal gyrus_anterior L | | | SupTepGyr_Mid_R | superior temporal gyrus_middle R |
| SupFrtGyr_Pst_L | superior frontal gyrus_posterior L | | | SupTepGyr_Pst_R | superior temporal gyrus_posterior R |
| SupTepGyr_Mid_L | superior temporal gyrus_middle L | | | SprmarGyr_Pst_R | supramarginal gyrus_posterior R |
| AnguGyr_Ant_L | angular gyrus_anterior L | | | SupPariGyr_Ant_R | superior parietal gyrus_anterior R |
| InfOcciGyr_Ant_L | inferior occipital gyrus_anterior L | | | SprmarGyr_Ant_R | supramarginal gyrus_anterior R |
| InfOcciGyr_VenPst_L | inferior occipital gyrus_ventroposterior L | | | ParsTriagu_Pst_R | pars triangularis_posterior R |
| TsvTepGyr_L | transverse temporal gyrus L | | | ParsOrbitalis_R | pars orbitalis R |
| ParsOpcu_Sup_L | pars opercularis_superior L | | |  |  |
| InfOcciGyr_DsoPst_L | inferior occipital gyrus_dorsoposterior L | | |  |  |
| SupTepGyr_Ant_L | superior temporal gyrus_anterior L | | |  |  |
| MidFrtGyr_Pst_L | middle frontal gyrus_posterior L | | |  |  |
| SprmarGyr_Pst_L | supramarginal gyrus_posterior L | | |  |  |
